# Real-World Data on the Associations of Tricyclic Antidepressants and Selective Serotonin Reuptake Inhibitors with Gynecologic Cancer Risk

**DOI:** 10.3390/cancers17101616

**Published:** 2025-05-10

**Authors:** Ching-Huan Wang, Chih-Wei Huang, Nhi Thi Hong Nguyen, Ming-Chin Lin, Phung-Anh Nguyen, Md. Mohaimenul Islam, Shuo-Chen Chien, Hsuan-Chia Yang

**Affiliations:** 1 Graduate Institute of Biomedical Informatics, College of Medical Science and Technology, Taipei Medical University, Taipei 110301, Taiwan; cwang226@alumni.jh.edu (C.-H.W.); gracehuang@tmu.edu.tw (C.-W.H.); arbiter@tmu.edu.tw (M.-C.L.); d610108001@tmu.edu.tw (S.-C.C.); 2 International Center for Health Information Technology (ICHIT), College of Medical Science and Technology, Taipei Medical University, Taipei 110301, Taiwan; 3 Health Personnel Training Institute, University of Medicine and Pharmacy, Hue University, Hue City, Vietnam; nthnhi@hueuni.edu.vn; 4 School of Nutrition and Health Sciences, College of Nutrition, Taipei Medical University, Taipei 110301, Taiwan; 5 Department of Neurosurgery, Taipei Municipal Wanfang Hospital, Taipei Medical University, Taipei 110301, Taiwan; 6 Graduate Institute of Data Science, College of Management, Taipei Medical University, Taipei 110301, Taiwan; alex0303@tmu.edu.tw; 7 Clinical Big Data Research Center, Taipei Medical University Hospital, Taipei 110301, Taiwan; 8 Research Center of Healthcare Industry Data Science, College of Management, Taipei Medical University, Taipei 110301, Taiwan; 9 Clinical Data Center, Office of Data Science, Taipei Medical University, Taipei 110301, Taiwan; 10 College of Pharmacy, University of Iowa, Iowa City, IA 52242, USA; mdmohaimenul-islam@uiowa.edu; 11 Research Center of Big Data and Meta-Analysis, Taipei Municipal Wanfang Hospital, Taipei Medical University, Taipei 110301, Taiwan

**Keywords:** tricyclic antidepressant, selective serotonin reuptake inhibitor, gynecologic cancer, cancer risk, real-world data

## Abstract

**Background**: While the potential anti-cancer effects of antidepressants have been investigated, limited research has been conducted incorporating age-specific analyses for individual tricyclic antidepressants (TCAs) and selective serotonin reuptake inhibitors (SSRIs). This study aims to elucidate the associations between TCAs and SSRIs with gynecologic cancers and to identify specific age groups and cancer types that may benefit from the chemopreventive effects of these medications. **Methods**: A case-control study comprised 97,736 female patients diagnosed with gynecologic cancers between 2002 and 2016. Each newly diagnosed case of cervical, ovarian, or uterine cancer was matched with four controls. Both cases and controls were then stratified by age to perform subgroup analyses. Associations between antidepressant use and cancer risk were evaluated using multivariable conditional logistic regression models. **Results**: The TCA class was significantly associated with reduced risks of cervical, ovarian, and uterine cancers, displaying adjusted odds ratios (aORs) of 0.799, 0.775, and 0.813, respectively. The SSRI class also indicated reduced risks, with aORs of 0.736, 0.638, and 0.567 for the same cancer types. Particularly noteworthy were females aged 40–64, who demonstrated the most significant associations between gynecologic cancers and using TCAs or SSRIs. **Conclusions**: TCAs and SSRIs are associated with reduced risks of developing cervical, ovarian, and uterine cancers. The middle-aged population may have the most significant potential for future research on drug repurposing against gynecologic cancers, and both cervical and uterine cancers are potential targets for drug repurposing involving TCAs or SSRIs.

## Simple Summary

Despite previous studies suggesting antidepressants’ potential anticancer effects against gynecologic cancers, little is known about these effects across different age groups. This study aims to investigate the associations of individual tricyclic antidepressants (TCAs) and selective serotonin reuptake inhibitors (SSRIs) with the risk of gynecologic cancers. We found that TCA and SSRI use was significantly associated with lowered risks of gynecologic cancers particularly among women aged 40–64. In contrast, no significant protective effect against ovarian or uterine cancer was observed in elderly women. We concluded that middle-aged women may benefit the most from future research on drug repurposing antidepressants for gynecologic cancer prevention.

## 1. Introduction

Depression is a significant factor contributing to global disability, ranking third for females and fifth for males in 2017 [1]. It is estimated that depressive disorders affect approximately 264 million people worldwide, accounting for 3–4% of the global population [1]. The lifetime prevalence of depression is reported to be 10.8%, with a higher susceptibility observed in females compared to males [2]. Since 2000, the consumption of antidepressants has doubled [3]. Antidepressants are primarily formulated to target amine signaling pathways under the monoamine-deficiency hypothesis of depression [4]. Pharmacologically, these agents can be classified into tricyclic antidepressants (TCAs), selective serotonin reuptake inhibitors (SSRIs), and monoamine oxidase inhibitors (MAOIs), among others [5]. Alongside the growing demand for antidepressant medications, concerns have emerged regarding drug safety and the potential for repurposing existing drugs.

Research exploring the association between antidepressant use and cancer risk has been ongoing for several decades. The first substantial study on this topic can be traced back to 1995, when Steingart et al. reported ambiguous findings on antidepressants’ dual potential for both promoting and inhibiting tumor growth, leaving the exact relationship inconclusive [6]. More recently, various hypotheses have been put forth to explain a potential association between antidepressants and gynecologic cancer risk, including increased release of gonadotropins and prolactin [7] as well as induced apoptosis of cancer cells [8]. However, empirical findings have been inconsistent. Some studies have suggested a potential risk reduction in ovarian [9] and endometrial [10] cancers with SSRI usage, whereas others have indicated an increased risk of ovarian cancer associated with TCAs [11]. Meanwhile, other research has reported that neither TCAs [7,10,12] nor SSRIs [7,12] show a significant association with ovarian [7], cervical [12], or endometrial cancers [10].

In Taiwan, the prevalence rates of uterine body, ovarian, and cervical cancers rank fifth, seventh, and ninth, respectively, among female cancers [13]. Previous observational studies have investigated the association between antidepressant use and the risk of gynecologic cancers; however, limited research has been conducted on age-specific effects related to individual TCAs or SSRIs. Therefore, our study aims to assess the chemopreventive potential of antidepressants with a focus on identifying age groups and types of gynecologic cancers that warrant further research. Utilizing real-world claims data, we examined the association between antidepressant use and the incidence of new cases of gynecologic cancers. Given that TCAs and SSRIs constitute the primary classes of antidepressants in our dataset and have the most extensive user base, we limited our focus to these two categories. We also stratified the subject population into three age subgroups to preliminarily observe variations in the impact of antidepressants on gynecologic cancers among users of different ages. The findings from this study may offer valuable insights for healthcare professionals and researchers interested in repurposing TCAs or SSRIs for the prevention of gynecologic cancers.

## 2. Methods

### 2.1. Study Design and Population

**Figure 1:**
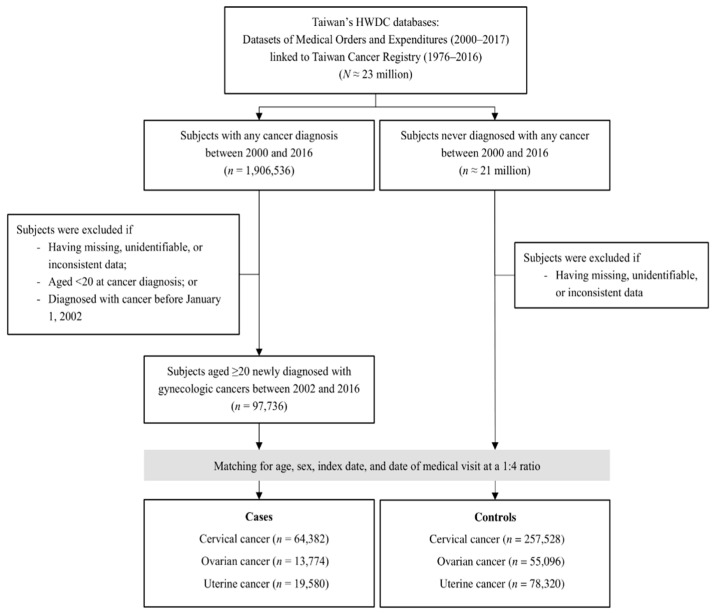
Flowchart of the study design.

This study employed a case-control design. We recruited patients with a new cancer diagnosis between 2000 and 2016 (Figure 1). Individuals were excluded from the analysis if their age at cancer diagnosis was under 20 or if their data were missing, unidentifiable, or inconsistent. Additionally, subjects initially diagnosed with cancer in 2000 or 2001 were excluded, as the study required verification of antidepressant use for at least two years prior to the index date. The index date for each case was defined as the date on which the cancer diagnosis was confirmed. Each control was assigned the same index date as its corresponding case. The study examined the association between the risk of cervical, ovarian, and uterine cancers and the usage of two classes of antidepressants: TCAs and SSRIs. Cases and controls were stratified into four age groups: young adults (20–39 years), middle-aged adults (40–64 years), elderly adults (≥ 65 years), and an overall age population (all subjects aged ≥  20). Subsequent analyses were conducted to assess the gynecologic cancer risks associated with using individual TCAs or SSRIs.

### 2.2. Data Sources

We utilized databases from Taiwan’s Health and Welfare Data Science Center (HWDC) that include medication and diagnosis information spanning 2000–2017 and cancer data from 1979 to 2016 (Figure 1). The HWDC operates as a centralized data repository overseen by Taiwan’s Ministry of Health and Welfare (MOHW). This repository securely houses de-identified claims data from Taiwan’s National Health Insurance (NHI) system [14]. The NHI system in Taiwan offers healthcare coverage to 99.9% of the population, encompassing over 23 million beneficiaries [15]. For this study, we accessed specific medical orders and expenditures datasets alongside the Taiwan Cancer Registry dataset containing pathologically confirmed cancer diagnoses. Disease categorization relied on the International Classification of Diseases, 9th Revision, Clinical Modification (ICD-9-CM) coding system [14,16].

### 2.3. Cases and Controls

Cases in this study were identified as patients who received an initial diagnosis of cervical, ovarian, or uterine cancer, as confirmed by the Taiwan Cancer Registry in 2002. These cancer diagnoses were validated through pathological assessment. We matched cases and controls in a 1:4 ratio, using criteria such as age, sex, index date, and date of medical visit [17]. Controls were defined as individuals who had never received a cancer diagnosis up to the end of 2016. Our analysis focused on the risk associated with specific gynecologic cancers, which are identified by their respective ICD-9-CM codes: cervical cancer (180.x), uterine cancer (182.x), and ovarian cancer (183.0).

### 2.4. Definitions of Exposure to Antidepressants

Exposure to antidepressants was defined as a prescription of these medications for at least 60 days within a two-year period preceding the index date. Based on this definition, exposure to TCAs and exposure to SSRIs were not mutually exclusive. Those who had used both TCAs and SSRIs were classified as exposed in both the TCA and SSRI groups, respectively. Antidepressants were classified according to the World Health Organization’s Anatomical Therapeutic Chemical (ATC) Classification system. This study specifically examined TCAs (N06AAxx) and SSRIs (N06ABxx) [5] due to their prevalence among users. The TCA category included imipramine (N06AA02), clomipramine (N06AA04), amitriptyline (N06AA09), doxepin (A06AA12), dosulepin (N06AA16), and maprotiline (N06AA21). The SSRI category comprised fluoxetine (N06AB03), citalopram (N06AB04), paroxetine (N06AB05), sertraline (N06AB06), fluvoxamine (N06AB08), and escitalopram (N06AB10) [5].

### 2.5. Potential Confounding Factors

The potential confounders considered in this study were age, Charlson Comorbidity Index (CCI) score [18], and the usage of metformin (ATC code: A10BA02), aspirin (ATC code: B01AC06), or statins (ATC codes: C10AA). CCI comorbidities, excluding cancers (Table 1), were deemed confounders if a patient had been diagnosed with them on two or more occasions. Likewise, the use of metformin [19,20,21], aspirin [22,23,24], and statins (Table 1) [21,25] for a minimum of 60 days within the two-year period preceding the index date was considered confounding, owing to their potential association with cancer risks.

### 2.6. Statistical Analysis

Descriptive statistics were used to describe the participants’ demographic characteristics. Continuous variables were presented as means and standard deviations; categorical variables were presented as numbers and percentages. The cancer risk associated with each medication or class was evaluated using the adjusted odds ratio (aOR) derived from multivariable conditional logistic regression. The significance level was set at α = 0.05 (two-sided). A two-sided *p*-value less than 0.05 was considered statistically significant. Data were analyzed using SAS 9.4 (SAS Institute Inc., Cary, NC, USA).

### 2.7. Ethics Approval Statement

The study protocol was approved by the Joint Institutional Review Board of Taipei Medical University, Taipei, Taiwan (TMU-JIRB N202003609).

## 3. Results

### 3.1. Participant Characteristics

Between 2000 and 2016, we identified 1,906,536 new cases of cancer. Of these, 97,736 individuals were newly diagnosed with gynecologic cancer at the age of 20 or older between 2002 and 2016. This cohort included 64,382 cases of cervical cancer, 13,774 of ovarian cancer, and 19,580 of uterine cancer (Figure 1). The mean ages at first diagnosis were 49.74 years for cervical cancer, 51.75 years for ovarian cancer, and 53.68 years for uterine cancer (Table 1). The middle-aged demographic was the most prevalent across all three cancer types, constituting 53.71%, 63.15%, and 75.50% of cervical, ovarian, and uterine cancer cases, respectively. Relative to the elderly group, the younger cohort displayed a higher proportion of cervical cancer cases (27.74% compared to 18.55%), a similar proportion of ovarian cancer cases (18.49% compared to 18.36%), and a lower proportion of uterine cancer cases (9.73% compared to 14.77%).

### 3.2. Associations of Gynecologic Cancers with the TCA and SSRI Classes

Table 2 presents the associations between gynecologic cancer risks and the use of TCAs and SSRIs. TCA and SSRI classes were associated with statistically significant reductions in the risk of gynecologic cancers. Specifically, TCAs demonstrated a decreased risk for cervical cancer (aOR = 0.799; 95% CI: 0.751, 0.850; *p* < 0.0001), ovarian cancer (aOR = 0.775; 95% CI: 0.681, 0.882; *p* = 0.0001), and uterine cancer (aOR = 0.813; 95% CI: 0.732, 0.903; *p* = 0.0001). Similarly, SSRIs were associated with reduced risks for cervical cancer (aOR = 0.736; 95% CI: 0.693, 0.782; *p* < 0.0001), ovarian cancer (aOR = 0.638; 95% CI: 0.559, 0.728; *p* < 0.0001), and uterine cancer (aOR = 0.567; 95% CI: 0.505, 0.636; *p* < 0.0001). In the age-stratified analysis, TCA use demonstrated significant reductions in cancer risks for cervical cancer in individuals aged ≥ 40, for ovarian cancer in those aged 40–64, and for uterine cancer in those aged < 65. However, there were no significant associations between TCA use and ovarian or uterine cancers in the older population. On the contrary, SSRIs consistently exhibited a reduced risk of cervical, ovarian, and uterine cancers across all age groups, except for uterine cancer in individuals aged ≥ 65. Table 3 summarizes the statistical significance of aORs for gynecologic cancers associated with each TCA and SSRI, stratified by age.

### 3.3. Associations of Gynecologic Cancers with Individual TCAs and SSRIs

**Figure 2:**
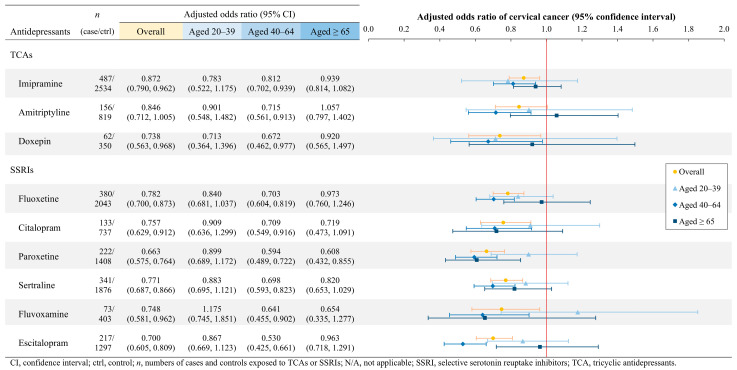
Cervical cancer risks associated with a single TCA or SSRI, stratified by age.

For cervical cancer, all individual TCAs and SSRIs, except for amitriptyline, were associated with a significant reduction in cancer risk (Figure 2). Age-specific analysis revealed that females between the ages of 20–39 exhibited no statistically significant association between cervical cancer risk and the use of TCAs or SSRIs. Conversely, females aged 40–64 who were treated with any TCA or SSRI showed a significantly decreased risk of developing cervical cancer. Among the elderly, paroxetine was the sole agent associated with a significant reduction in cervical cancer risk (aOR = 0.608; *p* = 0.0043).

**Figure 3:**
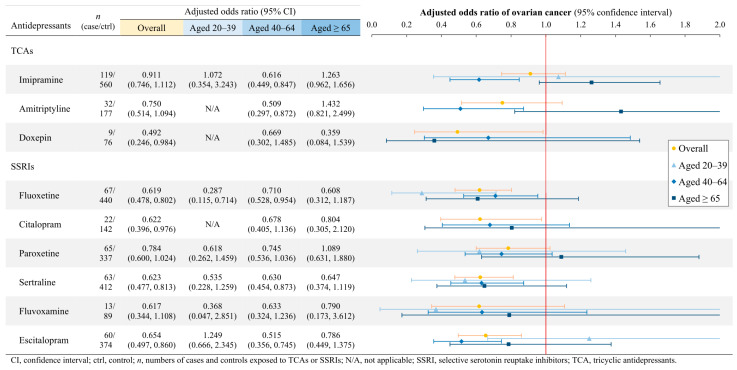
Ovarian cancer risks associated with a single TCA or SSRI, stratified by age.

Although the TCAs and SSRIs as classes were associated with significant reductions in ovarian cancer risk, the use of individual TCAs or SSRIs did not indicate a lowered risk of ovarian cancer (Figure 3). Across the general population, doxepin (aOR = 0.492; *p* = 0.0449), fluoxetine (aOR = 0.619; *p* = 0.0003), citalopram (aOR = 0.622; *p* = 0.0387), sertraline (aOR = 0.623; *p* = 0.0005), and escitalopram (aOR = 0.654; *p* = 0.0024) exhibited associations with decreased ovarian cancer risk. Notably, significant reductions in ovarian cancer risk were observed in 20-to-39-year-old users of fluoxetine (aOR = 0.287; *p* = 0.0073) and 40-to-64-year-old users of imipramine (aOR = 0.616; *p* = 0.0028), amitriptyline (aOR = 0.509; *p* = 0.0140), fluoxetine (aOR = 0.710; *p* = 0.0231), sertraline (aOR = 0.630; *p* = 0.0056), and escitalopram (aOR = 0.515; *p* = 0.0004). However, no significant reductions were observed in females aged 65 or older.

**Figure 4:**
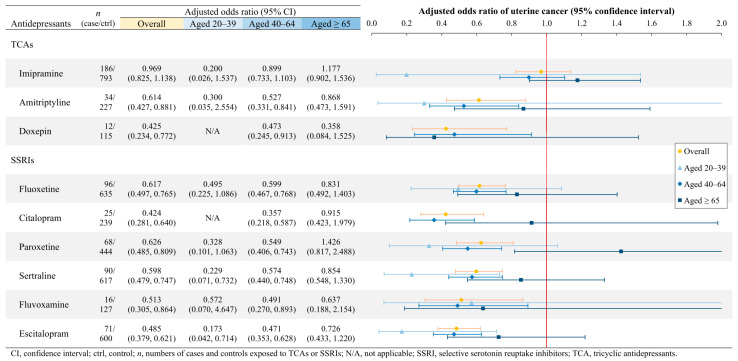
Uterine cancer risks associated with a single TCA or SSRI, stratified by age.

Regarding uterine cancer, the risk was generally reduced in all females treated with TCAs or SSRIs, as well as those aged 40–64, except imipramine users (Figure 4). In the younger age group, sertraline (aOR = 0.229; *p* = 0.0130) and escitalopram (aOR = 0.173; *p* = 0.0152) significantly reduced uterine cancer risk. In contrast, no individual TCA or SSRI exhibited a statistically significant association with uterine cancer risk in the elderly population.

## 4. Discussion

Our findings indicate a potential protective effect of TCAs and SSRIs against gynecologic malignancies, specifically in women aged 40–64 (Table 3). Both TCAs and SSRIs significantly reduced the risk of cervical cancer among users in this age group. In contrast, in the elderly population, only paroxetine was associated with a significant reduction in the risk of cervical cancer. Concerning ovarian cancer, specific agents—imipramine, amitriptyline, fluoxetine, sertraline, and escitalopram—were associated with a statistically significant reduction in cancer risk for women aged 40–64, while fluoxetine was effective in those aged 20–39. In the context of uterine cancer, all individual TCAs and SSRIs, except for imipramine, were associated with a significant reduction in risk for women aged 40–64. In younger individuals, however, this risk reduction was observed only in users of sertraline and escitalopram. Notably, no single TCA or SSRI was found to significantly mitigate the risks of ovarian and uterine cancers in elderly women.

In addition, our study revealed a clear age-dependent trend in antidepressant use. Among women aged 20–39 years, SSRIs were more frequently prescribed than TCAs, likely due to their more favorable safety and side-effect profiles. In the middle-aged group, the usage of TCAs and SSRIs was approximately balanced. Interestingly, in the elderly population, TCAs were used more frequently than SSRIs. This pattern reflects real-world prescribing practices in Taiwan, where SSRIs are preferred for younger patients, while TCAs remain common in older adults not only for long-standing depression but also for non-psychiatric indications such as neuropathic pain, including diabetic neuropathy and postherpetic neuralgia.

### 4.1. Comparison with Relevant Studies and Possible Mechanisms

#### 4.1.1. Cervical Cancer

To date, few studies have investigated the effects of antidepressants on the risk of cervical cancer. Our findings suggest that both TCA and SSRI classes are associated with a significantly decreased risk of cervical cancer. In contrast, a study conducted in Taiwan using the NHI Databases found no significant link between any major class of antidepressants and invasive cervical cancer [12]. The disparities in findings may be attributed to variations in study design, including the study’s duration, the study participants’ characteristics, and adjustments made for confounding variables. An in vitro study demonstrated that amitriptyline, a TCA, exerts cytotoxic effects on cervical cancer cells by inducing apoptosis, elevating reactive oxygen species levels, and inflicting irreversible mitochondrial damage [26]. Moreover, fluoxetine, an SSRI, has been shown to induce G0/G1 cell cycle arrest in cervical cancer cell lines and trigger apoptotic cell death by downregulating cyclin-dependent kinases regulatory subunit 1 (CKS1), a key protein in cell cycle regulation [27]. In our cohort, individuals aged 40–64 years who were using amitriptyline or fluoxetine exhibited a lower risk of cervical malignancy.

#### 4.1.2. Ovarian Cancer

The relationship between ovarian cancer risk and the use of TCA and SSRI has yielded inconsistent results in previous research. A Danish case-control study that utilized nationwide registries suggested a decreased risk of epithelial ovarian cancer in individuals taking SSRIs, particularly citalopram; however, no such association was observed in TCA users [9]. In alignment with these findings, our study also showed similar results for SSRIs and citalopram. Conversely, several other studies have failed to identify any correlation between antidepressant use and ovarian cancer risk [28,29,30,31,32]. Two meta-analyses that pooled data from multiple studies concluded that neither TCAs nor SSRIs significantly impact ovarian cancer risk [7,11]. One possible explanation for these conflicting findings may lie in the biphasic effect of antidepressants on cancer risk [7,11]. Low-dose antidepressant administration has been shown to stimulate malignant cell proliferation, while high-dose administration exhibits an inhibitory effect [7,11]. Our study’s findings of reduced risk might be partially attributed to our selection criteria, which required study participants to have used antidepressants for a minimum of 60 days within the two years preceding the index date. In vitro studies have supported these observations and shed light on the underlying mechanisms. For instance, imipramine, a TCA, was found to inhibit growth in the SK-OV-3 ovarian cancer cell line by blocking Ether-à-go-go (Eag) channels, voltage-gated potassium channels that suppress apoptosis in ovarian cancer cells [33]. Similarly, fluoxetine, an SSRI, was shown to induce cell death in SK-OV-3 and OVCAR-3 epithelial ovarian cancer cell lines by altering mitochondrial membrane permeability, triggering the release of cytochrome c, and activating caspase-3 [34]. At higher concentrations, fluoxetine also reduced the viability of ovarian granulosa tumor COV434 cell lines by affecting cytoplasmic Ca^2+^ accumulation and reducing intracellular cyclic AMP responses and ATP levels [35]. These in vitro observations may help elucidate our study results, which found a lower risk of ovarian cancer in imipramine users aged 40–64 and in fluoxetine users across all age groups except the elderly.

#### 4.1.3. Uterine Cancer

Limited research exists concerning the association between antidepressant use and uterine cancer risk. A case-control study focusing on Taiwanese cohorts categorized endometrial cancer patients into various subgroups according to their cumulative exposure to antidepressants [36]. This study found no significant association across subgroups with exposure to either SSRIs or TCAs. In contrast, our study revealed that both TCAs and SSRIs were associated with a significantly reduced risk of uterine cancer across all age categories, including younger and middle-aged groups. An in vitro investigation has indicated that amitriptyline significantly diminishes the viability of HTB114 uterine leiomyosarcoma cells [37]. This is accomplished by upregulating the p75 neurotrophin receptor (p75NTR), a tumor necrosis factor (TNF) receptor superfamily member. This upregulation leads to the downregulation of neurotrophin-selective tropomyosin receptor kinase (TrKA^+^)-prosurvival signaling, ultimately inducing p75^NTR^-dependent apoptosis via caspase-3 [37]. Our study also identified a considerable reduction in uterine cancer risk among both the overall age group and those aged 40 to 64 years. The disparities between our findings and previous research could be ascribed to differences in the study populations and the adjusted confounders.

#### 4.1.4. Elderly Population

Our results showed that most individual TCAs and SSRIs were associated with a significantly reduced risk of the three gynecologic cancers in women aged 40–64, but not among the elderly population, except for paroxetine in cervical cancer (Table 3). This might partly be explained by hormonal changes and immunosenescence in elderly women.

Pharmacologically, both TCAs and SSRIs increase serotonin activity by inhibiting serotonin reuptake [38,39]. An increased serotonin level can induce the secretion of prolactin [40] and estrogen [41,42]. Although previous research has demonstrated that prolactin and sex hormones are associated with an increased risk of gynecologic cancers [43,44,45,46], TCAs and SSRIs may exert additional hormonal regulatory mechanisms that could mitigate the pro-tumor influences of prolactin and estrogen, thereby contributing to a net antitumor effect. For instance, fluoxetine and sertraline can exhibit weak estrogenic or antiestrogenic effects depending on the concentration [47]. Fluoxetine has also been reported to downregulate estrogen receptor expression in vitro [48,49], while its effect on estradiol levels varies across animal models [49]. Sertraline and paroxetine enhance estradiol-mediated transcription at low concentrations [47], whereas paroxetine reduces estradiol levels dose-dependently [49]. Amitriptyline may improve ovarian morphology and normalize estradiol levels in rats with polycystic ovarian syndrome [49]. Thus, if the potential chemoprotective effects of TCAs and SSRIs against gynecologic cancers are mediated through hormonal modulation in middle-aged women, then these effects can be diminished in postmenopausal women due to the typically low baseline levels of these hormones.

Beyond hormonal factors, immunosenescence may outweigh the potential chemoprotective effect of TCAs and SSRIs in the elderly population. Age-related impairment of immune surveillance in elderly women weakens the ability to detect and eliminate precancerous cells. Interestingly, some antidepressants may have immunomodulatory effects. Fluoxetine has been shown to enhance cytotoxic T cell responses by reducing programmed cell death-ligand 1 (PD-L1) expression on tumor cells through the depletion of peripheral serotonin [50]. TCAs and SSRIs might lower the risks of gynecologic cancers, but this effect may be attenuated by immunosenescence in elderly women.

### 4.2. Interpretation of Our Findings for Future Research

The primary objective of this study is to identify associations, rather than establish causality, between antidepressant use and gynecologic cancers. We aim to provide comprehensive data findings that could enrich the existing evidence base and provide a robust foundation for subsequent research. Our data indicate associations of reduced cancer risk with both TCA and SSRI classes, thereby suggesting these medications as potential candidates for further scrutiny in cancer prevention efforts. Previous studies have highlighted multiple beneficial activities of antidepressants that could be leveraged for cancer treatment [51,52]. Considering that existing literature predominantly employs either in vitro or observational study designs [7,9,11,26,27], more comprehensive studies grounded in large-scale, real-world data are essential for gaining nuanced insights into the causal mechanisms between gynecologic cancers and antidepressant use. Our findings reveal that the middle-aged demographic exhibits the most promising potential for drug repurposing. When evaluating individual TCAs or SSRIs, it is noteworthy that the elderly group did not show a statistically significant association with gynecologic cancer risks for any single medication, with the exception of reduced cervical cancer risk related to paroxetine. This absence of significance could be attributed either to the ineffectiveness of TCAs and SSRIs in chemoprevention due to hormonal changes or immunosenescence among older females or to the limited sample size compared to the middle-aged group. Likewise, the limited number of participants in certain subgroups, particularly among young women, may reduce the statistical power for subgroup analyses and lead to statistically non-significant findings even if true associations exist. Furthermore, in middle-aged women, almost all individual medications were associated with significant reductions in both cervical and uterine cancer risks. As such, these two types of cancer may serve as prospective targets in future research focused on the repurposing of TCAs and SSRIs for chemoprevention. In addition, exposures were treated independently for each antidepressant, irrespective of concurrent or sequential use of multiple agents in an individual participant. Future research could investigate differences in chemoprotective effects between users of a single antidepressant and those using multiple agents, as well as the influence of concurrent versus sequential use of multiple antidepressants.

### 4.3. Limitations

This study has several limitations that warrant discussion. First, the research design allows us to identify associations, not causality, between individual antidepressants and cancer risks, calling for additional studies to elucidate underlying mechanisms [53,54]. Secondly, information about patients’ lifestyles, medication adherence, and laboratory data may not be available from the HWDC databases. However, the impact of this missing data is mitigated by verifying all cancer diagnoses through pathology reports and the substantial sample size.

Despite these limitations, our research has notable strengths rooted in high-quality registry data. Medical prescriptions are required for antidepressant medications in Taiwan, ensuring their comprehensive inclusion in the HWDC databases. Moreover, cancer diagnoses were rigorously verified using the Taiwan Cancer Registry database, confirming each diagnosis through pathology reports. Lastly, our extensive database, encompassing records from approximately 23 million patients, enabled age-based subject stratification and provided for a thorough analysis of the effects of individual TCAs and SSRIs.

## 5. Conclusions

Both TCAs and SSRIs are associated with significantly reduced risks of cervical, ovarian, and uterine cancers. The middle-aged demographic presents the most promising avenue for future research on drug repurposing against gynecologic cancers. Specifically, cervical and uterine cancers emerge as potential targets for repurposing individual TCAs or SSRIs.

To our knowledge, this is the first study utilizing extensive real-world data that includes age subgroup analysis for individual antidepressant agents against various types of cancers. Our findings underscore the promise of both TCA and SSRI classes in repurposing medications to prevent gynecologic cancers. They also advocate for future interventional research, such as randomized controlled trials, to clarify TCAs and SSRIs’ precise antineoplastic mechanisms and efficacy against these cancers. This study serves as the empirical foundation for generating hypotheses for future animal studies.

## Figures and Tables

**Table 1:** Demographic characteristics.

Characteristics	Cervical Cancer				Ovarian Cancer				Uterine Cancer			
	Cases (*n* = 64,382)		Controls (*n* = 257,528)		Cases (*n* = 13,774)		Controls (*n* = 55,096)		Cases (*n* = 19,580)		Controls (*n* = 78,320)	
**Age**												
Mean ± SD	49.74	± 14.48	49.74	± 14.48	51.72	± 13.96	51.72	± 13.95	53.68	± 10.93	53.68	± 10.92
20–39 years, *n* (%)	17,861	(27.74)	71,444	(27.74)	2547	(18.49)	10,188	(18.49)	1905	(9.73)	7620	(9.73)
40–64 years, *n* (%)	34,581	(53.71)	138,324	(53.71)	8698	(63.15)	34,792	(63.15)	14,783	(75.50)	59,132	(75.50)
≥65 years, *n* (%)	11,940	(18.55)	47,760	(18.55)	2529	(18.36)	10,116	(18.36)	2892	(14.77)	11,568	(14.77)
**Comorbid conditions, *n* (%)**												
Myocardial infarction	108	(0.17)	520	(0.20)	39	(0.28)	112	(0.20)	47	(0.24)	142	(0.18)
Congestive heart failure	896	(1.39)	4222	(1.64)	235	(1.71)	914	(1.66)	342	(1.76)	1195	(1.53)
Peripheral vascular disease	377	(0.59)	1884	(0.73)	97	(0.70)	405	(0.74)	128	(0.65)	643	(0.82)
Cerebrovascular disease	2347	(3.65)	11,388	(4.42)	545	(3.96)	2560	(4.65)	831	(4.24)	3518	(4.49)
Dementia	437	(0.68)	2409	(0.94)	102	(0.74)	497	(0.90)	88	(0.45)	521	(0.67)
Chronic pulmonary disease	1766	(2.74)	8427	(3.27)	370	(2.69)	1760	(3.19)	544	(2.78)	2478	(3.16)
Rheumatic disease	898	(1.39)	4141	(1.61)	219	(1.59)	935	(1.70)	246	(1.26)	1396	(1.78)
Peptic ulcer disease	6348	(9.86)	31,919	(12.39)	1814	(13.17)	7020	(12.74)	2083	(10.64)	10,536	(13.45)
Liver disease	2909	(4.52)	15,551	(6.04)	893	(6.48)	3420	(6.21)	1256	(6.41)	5337	(6.81)
Diabetes	6452	(10.02)	34,379	(13.35)	1664	(12.08)	8312	(15.09)	3506	(17.91)	12,780	(16.32)
Hemiplegia or paraplegia	103	(0.16)	481	(0.19)	30	(0.22)	140	(0.25)	24	(0.12)	149	(0.19)
Renal disease	1272	(1.98)	5543	(2.15)	281	(2.04)	1322	(2.40)	466	(2.38)	2015	(2.57)
AIDS/HIV infection	38	(0.06)	42	(0.02)	0	(0.00)	9	(0.02)	0	(0.00)	13	(0.02)
**CCI scores**												
Mean (SD) *	0.40	(0.87)	0.50	(0.92)	0.49	(0.94)	0.53	(0.94)	0.53	(0.94)	0.56	(0.94)
**Other drugs, *n* (%)**												
Metformin	3990	(6.20)	23,451	(9.11)	1192	(8.65)	6186	(11.23)	2408	(12.30)	10,291	(13.14)
Aspirin	3775	(5.86)	19,109	(7.42)	906	(6.58)	4436	(8.05)	1545	(7.89)	6401	(8.17)
Statin	3708	(5.76)	20,402	(7.92)	943	(6.85)	5125	(9.30)	2162	(11.04)	7891	(10.08)

AIDS, acquired immunodeficiency syndrome; HIV, human immunodeficiency virus; *n*, number; *N*, total number; SD, standard deviation. * All CCI scores are ≥0. Due to the right-skewed distribution of comorbidities, mean ± SD may appear to allow negative values, but no negative values are present in the dataset.

**Table 2:** Risk of gynecologic cancers associated with the TCA and the SSRI classes, stratified by age.

	Cervical Cancer (64,382 Cases; 257,528 Controls)			Ovarian Cancer (13,774 Cases; 55,096 Controls)			Uterine Cancer (19,580 Cases; 78,320 Controls)		
Antidepressants	*n* (Case/ Ctrl)	Crude OR (95% CI)	Adjusted OR ^a^ (95% CI)	*n* (Case/ Ctrl)	Crude OR (95% CI)	Adjusted OR ^a^ (95% CI)	*n* (Case/ Ctrl)	Crude OR (95% CI)	Adjusted OR ^a^ (95% CI)
**TCAs**									
Overall	1249/ 6800	0.729 (0.686, 0.775)	0.799 (0.751, 0.850)	282/ 1521	0.736 (0.647, 0.837)	0.775 (0.681, 0.882)	431/ 2177	0.787 (0.709, 0.874)	0.813 (0.732, 0.903)
Aged 20–39	143/ 681	0.839 (0.700, 1.005)	0.876 (0.730, 1.050)	10/ 66	0.605 (0.310, 1.177)	0.605 (0.310, 1.179)	6/ 66	0.362 (0.157, 0.835)	0.327 (0.140, 0.765)
Aged 40–64	618/ 3731	0.656 (0.602, 0.715)	0.740 (0.678, 0.807)	146/ 961	0.601 (0.504, 0.716)	0.655 (0.549, 0.782)	274/ 1576	0.690 (0.606, 0.785)	0.724 (0.636, 0.825)
Aged ≥ 65	488/ 2388	0.810 (0.733, 0.894)	0.867 (0.784, 0.959)	126/ 494	1.021 (0.836, 1.248)	1.015 (0.830, 1.242)	151/ 535	1.136 (0.944, 1.367)	1.132 (0.940, 1.363)
**SSRIs**									
Overall	1262/ 7154	0.700 (0.659, 0.743)	0.736 (0.693, 0.782)	262/ 1656	0.626 (0.549, 0.714)	0.638 (0.559, 0.728)	339/ 2412	0.554 (0.494, 0.622)	0.567 (0.505, 0.636)
Aged 20–39	355/ 1650	0.858 (0.764, 0.963)	0.878 (0.782, 0.987)	28/ 202	0.550 (0.369, 0.818)	0.553 (0.371, 0.825)	15/ 194	0.304 (0.179, 0.515)	0.313 (0.184, 0.530)
Aged 40–64	627/ 4068	0.609 (0.560, 0.663)	0.649 (0.596, 0.707)	176/ 1136	0.612 (0.521, 0.718)	0.641 (0.545, 0.753)	244/ 1863	0.516 (0.451, 0.590)	0.531 (0.464, 0.608)
Aged ≥ 65	280/ 1436	0.775 (0.680, 0.882)	0.816 (0.716, 0.930)	58/ 318	0.723 (0.545, 0.960)	0.699 (0.525, 0.930)	80/ 355	0.899 (0.703, 1.149)	0.897 (0.700, 1.149)

^a^ Adjusted for age, the Charlson Comorbidity Index, and use of medications (metformin, aspirin, and statins). CI, confidence interval; ctrl, control; n, number of both cases and controls exposed to TCAs or SSRIs; OR, odds ratio; SSRIs, selective serotonin reuptake inhibitors; TCAs, tricyclic antidepressants.

**Table 3:** Summary of significantly lowered gynecologic cancer risks associated with TCAs or SSRIs.

	Cervical Cancer				Ovarian Cancer				Uterine Cancer			
Age Groups	Overall	20–39	40–64	≥65	Overall	20–39	40–64	≥65	Overall	20–39	40–64	≥65
**TCAs**	****		****	**	***		****		***	**	****	
Imipramine	**		**				**					
Amitriptyline			**				*		**		**	
Doxepin	*		*		*				**		*	
**SSRIs**	****	*	****	**	****	**	****	*	****	****	****	
Fluoxetine	****		****		***	**	*		****		****	
Citalopram	**		**		*				****		****	
Paroxetine	****		****	**					***		***	
Sertraline	****		****		***		**		****	*	****	
Fluvoxamine	*		*						*		*	
Escitalopram	****		****		**		***		****	*	****	

*p*-values of adjusted odds ratios: * *p* < 0.05; ** *p* < 0.01; *** *p* <0.001; **** *p* < 0.0001. SSRIs, selective serotonin reuptake inhibitors; TCAs, tricyclic antidepressants.

## Data Availability

The datasets developed and used in the present study are not publicly accessible, but they are available the Taiwan’s Ministry of Health and Welfare upon reasonable request.
